# Editorial Correspondence

**Published:** 1877-06

**Authors:** W. H. Hardison

**Affiliations:** Larned, Kans.


Larned, Kans., May 25, 1877.

*Editors Atlanta Medical and Surgical Journal:*

I was very much interested in the communication in the last number of your journal, from Dr. W. A. Adams, of Bryan, Texas, concerning an epidemic of roseola. Though nearly 800 miles north of Bryan, and in quite a different climate, yet for two months we have had a similar epidemic in this city. The eruption generally extends from crown to sole, and the diameters of the circles vary from one-eighth to one-half of an inch. Itching is, almost without exception, from the appearance to the final disappearance, a prominent feature. The eruption generally lasts from three to five days. The tonsils, submaxillary and lymphatic glands are generally enlarged, as in the cases of Dr. Adams, and deglutition more or less painful. While it has not appeared so prevalent in adults as in children, it has been more stubborn in the former. Out of perhaps twenty who have been under my personal observation and professional care, not one has died; yet a few deaths have occurred from what the attending physicians pronounced measles and scarlatina.

My treatment has been intended—after regulating the secretions, which are generally scanty, by giving a dose of calomel at the beginning—to keep the bowels open with mild laxatives, such as cream tarter and sulphur, and to keep the patient more or less under the influence of opium; preferring Dover's powder, on account of the ipecac contained therein. As to the "anasarca" which Dr. A. spoke of, I have not witnessed a single case in my practice, nor have I heard any doctor in the city speak of it, in which the cause was attributed to a previous attack of the skin disease.

Now, why do ten per cent. of those who suffer from the disease in Bryan have dropsy as a sequel, while here we are entirely rid of it? Is the disease different? With the exceptions above mentioned, the symptoms are the same as described by Dr. Adams. Is it due to difference in climate? It is much colder here, and the atmosphere drier and lighter than in Bryan. Or is it due to difference in treatment? though our treatment was not intended as anti-dropsical or in anticipation of same. Is roseola prevalent over the country as an epidemic?

Respectfully, W. H. HARDISON, M.D.

